# Spectral Properties of Substituted Coumarins in Solution and Polymer Matrices 

**DOI:** 10.3390/molecules17033259

**Published:** 2012-03-14

**Authors:** Jana Donovalová, Marek Cigáň, Henrieta Stankovičová, Jan Gašpar, Martin Danko, Anton Gáplovský, Pavol Hrdlovič

**Affiliations:** 1 Faculty of Natural Sciences, Institute of Chemistry, Comenius University, Mlynská dolina CH-2, SK-842 15 Bratislava, Slovak Republic; Email: cigan@fns.uniba.sk (M.C.); stankovh@fns.uniba.sk (H.S.); gaspar@fns.uniba.sk (J.G.); gaplovsky@fns.uniba.sk (A.G.); upolhrdl@savba.sk (P.H.); 2 Polymer Institute, Slovak Academy of Sciences, 845 41 Bratislava, Dúbravská cesta 9, Slovak Republic; Email: upoldan@savba.sk

**Keywords:** fluorescence, substituted coumarins, solvent effect, polymer matrices

## Abstract

The absorption and fluorescence spectra of substituted coumarins (2-oxo-2*H*-chromenes) were investigated in solvents and in polymer matrices. The substitutions involved were: (1) by groups with varying electron donating ability such as CH_3_, OCH_3_ and N(CH_3_)_2_, mainly, but not exclusively, in positions 7 and (2), by either CHO or 4-PhNHCONHN=CH- in position 3. While the spectra of non-substituted coumarin-3-carbaldehyde has absorptions at approximately 305 and 350 nm, substitution at position 7 leads to remarkable changes in the shape of the absorption spectrum and shifts the absorption to a longer wavelength. Similarly, the replacement of the formyl group with a semicarbazide group substantially influences the shape of the absorption spectrum, and coumarins which have only N(CH_3_)_2_ in position 7 experience small changes. These changes are associated with the increasing intramolecular charge transfer (ICT) character and increasing conjugation length of the chromophoric system, respectively, in the studied molecules. The fluorescence is almost negligible for derivatives which have H in this position. With increasing electron donating ability, and the possibility of a positive mesomeric (+M) effect of the substituent in position 7 of the coumarin moiety, the fluorescence increases, and this increase is most intense when N(CH_3_)_2_ substitutes in this position, for both 3-substituted derivatives. Spectral measurements of the studied coumarins in polymer matrices revealed that the absorption and fluorescence maxima lay within the maxima for solvents, and that coumarins yield more intense fluorescence in polymer matrices than when they are in solution. The quantum yield of derivatives which have a dimethylamino group in position 7 in polymer matrices approaches 1, and the fluorescence lifetime is within the range of 0.5–4 ns. The high quantum yield of 7-dimethylamino derivatives qualifies them as laser dyes which have *k*_F_ higher than *k*_nr_ in the given medium. This is caused by stiffening of the coumarin structure in polar polymer matrices, such as PMMA and PVC, due to higher micro-viscosity than in solution and intermolecular dipole-dipole interaction between chromophore (dopant) and matrix.

## 1. Introduction

Although the non-substituted parent coumarin (2-oxo-2*H*-chromene) exhibits zero or very weak fluorescence, properly substituted derivatives yield intense fluorescence and these are widely used in different branches of chemistry, biology, medicine and physics [1,2]. These derivatives are an important part of fluorescence probes, sensors and switches, as reviewed by Prasanna de Silva [3,4]. These have also been recently used for enantioselective sensing [5].

The influence of the environment (proximity) of substituted coumarins on photo-physics has been extensively studied with steady state and time resolved spectroscopy [5,6,7,8,9,10,11,12,13,14,15,16,17,18,19,20,21]. Since the fluorescence of coumarin derivatives is dependent on their environment, they are an important structural unit for probes widely used in monitoring the polarity and micro-viscosity of the environment in various simple, mixed or ionic solvents [1,2,3,4,5,6,7,8,9,10,11,12,13,14,15,16,17,18,19,20,21].

The strong solvent dependence of various coumarins has been exploited in the characterization of micelles [22,23,24,25]. They respond spectrally to other cavities, including inclusion complexes [26,27], within the cavities of porous materials and on their surface [28,29]. They are used in the characterization of materials prepared by sol-gel processes [30,31,32], and of nano-particles such as silica and silver [33,34].

The fluorescence of coumarins is widely used as a research tool in polymer science [35,36]. Moreover, they are used as photo-initiators [37], for incorporation into polymer chains by co-polymerization [38], in the estimation of polymer solvent effects [39,40,41], for various structural characterization [42,43], in the monitoring of the releasing properties of poly(methylmethacrylate) nanospheres [44] and for polymeric fluorescent solar collectors [45]. The influence of the polymer matrix on the decay of fluorescence of dialkylamino-substituted coumarins [46] and on the properties of photo-responsive hyperbranched polyesters [47] has been studied systematically. Coumarins may also serve as model compounds for lignin [48] and the characterization of cellulose surface polarity [49].

The quenching of 7-amino substituted coumarins by diphenyl and triphenylamine derivatives has been investigated by both steady state and time resolved spectroscopy [50,51]. Therein, it was concluded that electron transfer in this process fits within the framework of the Marcus electron transfer theory.

Coumarins have been used as structural units in fluorescence probes based on intramolecular quenching, and as reporters of radical reactions within solutions and thin polymer films [52,53,54]. The extent of intramolecular quenching depends on the coumarin structure, and for simple coumarin this was established to be in the range of 2–5 in solution and up to 20 in polymer matrices [54].

Together with other chromophores, substituted coumarins are used as molecular rotors and fluorescence probes in biological studies [55].

Optical properties of the laser dye coumarin 500 were investigated after exposure to atmospheric coronal discharge, in order to imitate the degradation occurring during their application as a laser or textile dye. When this discharge exposure was found to result in extensive degradation [56], a new molecular design for coumarin fluorescence dyes was created. This was achieved by the introduction of an arylsulfonated group, and this improved its application in the textile industry [57].

Improved performance of probes and sensors based on coumarin chromophores was achieved by introducing an additional structural unit like a rigid crown to improve ion selectivity [58]. The exploitation of energy transfer was effected by introducing an additional chromophore, such as phthalimide [59] or 1,8-naphthylimide [60,61].

Those coumarins substituted by dialkylamino and/or amino goups are widely studied because of their ability to yield intense fluorescence or lase in the blue-green region. Their photo-physical properties are modified by electron withdrawing substituents, such as CHO or –CH=NNHCONHPh [62] in position 3. This study reveals that the 3-carbaldehyde exhibits a strong solvent effect in polar solvents due to the formation of a non-fluorescent twisted intramolecular charge transfer (TICT) state, while a locally excited intramolecular charge transfer (LE/ICT) state prevails when –CH=NNHCONHPh substitutes in position 3.

**Figure 1:**
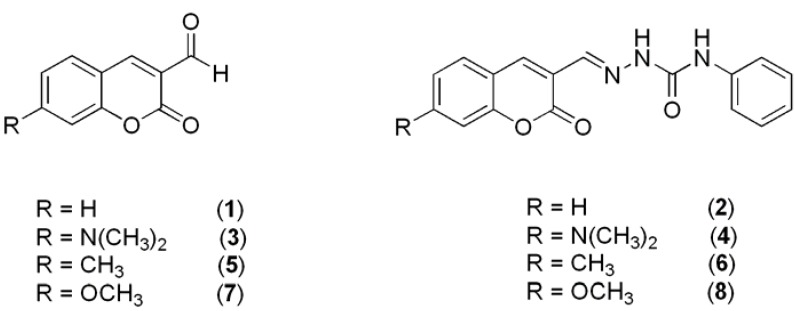
Molecular structure of the studied molecules.

In this study, eight coumarin derivatives [2-oxo-2*H*-chromene-3-carbaldehyde (1), (*E*)-1-[(2-oxo-2*H*-chromen-3-yl)methylidene]-4-phenylsemicarbazide (**2**), 7-(dimethylamino)-2-oxo-2*H*-chromene-3-carbaldehyde, (**3**), (*E*)-1-{[(7-dimethylamino)-2-oxo-2*H*-chromen-3-yl]methylidene}-4-phenyl-semicarbazide (**4**), 7-methyl-2-oxo-2*H*-chromene-3-carbaldehyde (**5**), (*E*)-1-[(7-methyl-2-oxo-2*H*-chromen-3-yl)methylidene]-4-phenylsemicarbazide (**6**), 7-methoxy-2-oxo-2*H* chromene-3-carbaldehyde (**7**) and (*E*)-1-[(7-methoxy-2-oxo-2*H*-chromen-3-yl)methylidene]-4-phenylsemicarbazide **8 **(Figure 1)], substituted in position 3 with an electron withdrawing substituent and with substituents of varying electron donating ability in position 7 were investigated spectrally in solution and in a polymer matrix.

Two types of substituents in position 3, namely CHO and PhNHCONHN=CH, were compared regarding the ability to modify their spectral properties by binding various other units through hydrogen bonding. The steady state and time-resolved fluorescence measurements were performed in aerated solutions. All measurements on polymer films were performed in the air.

## 2. Results and Discussion

### 2.1. Absorption Spectra

**Figure 2:**
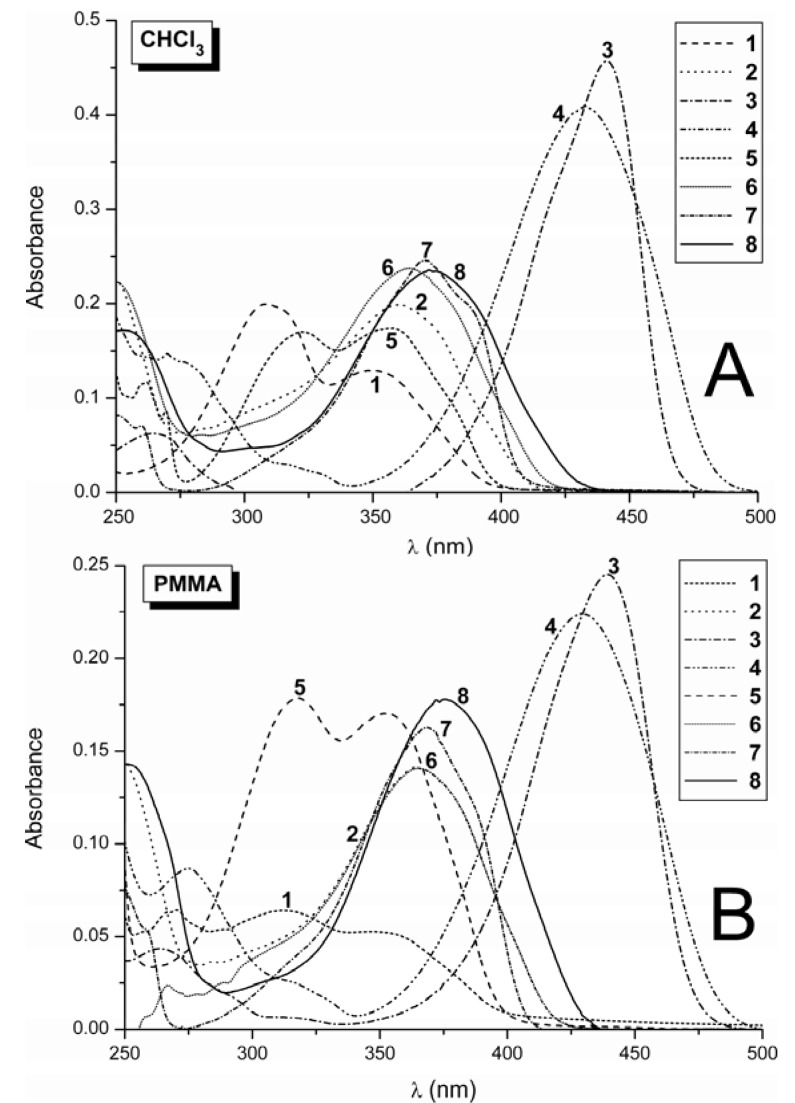
(**A**) Absorption spectra of coumarins **1**–**8** in chloroform at 10^−5^ mol·dm^−3^. (**B**) Absorption spectra of coumarins **1**–**8** in PMMA at 0.002 mol·kg^−1^.

The aim of the spectral study of the studied coumarins with electron withdrawing substituent like CHO or CH=N-NHCONHPh in position 3 and electron donating substituents with increasing electron donating power in position 7 (leading to formation of a push-pull system on coumarin structural unit) was to gain some insight into their photo-physics. The absorption spectra of the studied derivatives are quite similar in solution (Figure 2A) and in the polymer matrices (Figure 2B). The relevant absorption data established in the different media for derivatives **1**–**8** are summarized in Table 1,Table 2 and Table 3.

**Table 1:** Spectral data on UV absorption spectra of **1**, **2**, **5**, **6** and **7**.

Comp./Medium	1 *λ*_A_ / log *ε*(nm)	2 *λ*_A_ / log *ε*(nm)	5 *λ*_A_ / log *ε*(nm)	6 *λ*_A_ / log *ε*(nm)	7 *λ*_A_ / log *ε*(nm)
MeOH	278 / 3.78 *		287 / 4.37 *		
MeOH	310 / 3.56 *		315 / 4.32 *		
MeOH	354 / 3.10 *	359 / 4.30	360 / 4.03 *	361 / 4.41	361 / 3.96 *
CHCl_3_	307 / 4.30		321 / 4.27		
CHCl_3_	348 / 4.12	360 / 4.30	356 / 4.24	364 / 4.37	370 / 4.39
PMMA	306 / 3.90		312 / 4.31		
PMMA	351 / 3.72	361 / 4.15	347 / 4.23	364 / 4.15	368 / 4.20
PVC	311 / 3.85		321 / 4.12		
PVC	357 / 3.67	364 / 4.24	357 / 4.08	368 / 4.26	372 / 4.32
PS	314 / 3.85		323 / 4.06		
PS	351 / 3.74	365 / 4.11	357 / 4.07	365 / 4.21	372 / 4.22

**Scheme 1:**
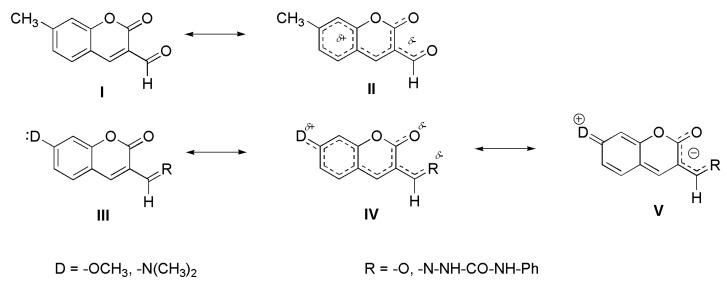
Proposed intramolecular charge transfer (ICT) character of the studied molecules.

The structure of compounds is given in Scheme 1; *λ*_A_: maximum of absorption, log *ε*: log of decadic extinction coefficient; *: distorted value due to the formation of acetal.

**Table 2:** Spectral properties of disubstituted derivatives of coumarin **3**, **4** and **8**.

Comp./Medium	*λ*_A_ / log *ε*(nm)	*Δν*_1/2_(cm^−1^)	*λ*_F_(nm)	*ν*_A_–*ν*_F_(cm^−1^)	Φ_F_	*τ*(ns)	*k*_F_(10^9^ s^−1^)	*k*_nr_(10^9^ s^−1^)
**3**								
MeOH	436 / 4.49	3355	492	2611	0.40 ± 0.06	1.1 ± 0.1	0.37	0.54
CHCl_3_	441 / 4.66	2580	470	1399	0.44 ± 0.07	2.6 ± 0.1	0.17	0.22
PMMA	440 / 4.39	2939	475	1675	0.81 ± 0.12	3.3 ± 0.2	0.24	0.06
PVC	446 / 4.56	2918	482	1675	1.08 ± 0.16	4.0 ± 0.2	0.27	-
PS	438 / 4.41	2555	468	1464	0.22 ± 0.03	2.5 ± 0.1	0.09	0.31
**4**								
MeOH	431 / 4.48	3402	503	3321	0.64 ± 0.10	1.8 ± 0.1	0.36	0.20
CHCl_3_	433 / 4.61	3811	492	2769	0.49 ± 0.07	1.8 ± 0.1	0.27	0.28
PMMA	432 / 4.35	3894	504	3307	1.08 ± 0.16	3.6 ± 0.2	0.30	-
PVC	440 / 4.43	3805	505	2925	1.10 ± 0.17	2.3 ± 0.1	0.48	-
PS	429 / 4.42	4437	505	3508	0.25 ± 0.04	2.6 ± 0.1	0.10	0.29
**8**								
MeOH	373 / 4.54	5431	474	5713	0.007 ± 0.001			
CHCl_3_	375 / 4.37	5343	464	4973	0.011 ± 0.002	3.3 ± 0.2	0.003	0.30
PMMA	375 / 4.25	4842	463	5068	0.30 ± 0.05	2.3 ± 0.1	0.13	0.30
PVC	379 / 4.38	4547	466	4926	0.29 ± 0.04	1.9 ± 0.1	0.15	0.37
PS	375 / 4.14	4876	465	5161	0.06 ± 0.01	0.7 ± 0.04	0.09	1.34

Structure of the coumarins is given in Scheme 1. The media are as follows: methanol: MeOH, Chloroform: CHCl_3_, poly(methyl metharylate): PMMA, poly(vinylchloride): PVC, polystyrene: PS; *λ*_A_: maximum of absorption, log *ε*: log of decadic extinction coefficient, Δ*ν*_1/2_: half width of the longest wavelength absorption band *λ*_F_: maximum of fluorescence, *ν*_A_–*ν*_F_: Stoke’s shift, Φ_F_: quantum yield of fluorescence based on anthracene,*τ*: experimental life time, *k*_F_: fluorescence rate constant from the S_1_ given as *k*_F_ = Φ/*τ*, *k*_nr_: radiationless rate constant from S_1_ given as *k*_nr_ = (1 − Φ)/*τ*.

**Table 3:** Calculated spectral properties of **3**, **4** and **8**, based on spectral data.

Comp./Medium	*E*_S1_(kJ mol^−1^)	*f*	*τ*_0_(ns)	*τ*_F-calc_(ns)	*k*_F-calc_(10^9^ s^−1^)	*k*_nr-calc_(10^9^ s^−1^)
**3**						
MeOH	258	0.33	8.5	3.4	0.12	0.18
CHCl_3_	263	0.34	8.6	3.8	0.12	0.15
PMMA	261	0.31	9.3	7.5	0.11	0.03
PVC	258	0.46	6.5	7.0	0.15	-
PS	264	0.29	1.0	0.2	0.99	3.57
**4**						
MeOH	256	0.34	8.3	5.3	0.12	0.07
CHCl_3_	259	0.45	6.2	3.0	0.16	0.17
PMMA	267	0.38	7.4	8.0	0.14	-
PVC	253	0.44	6.6	7.3	0.15	-
PS	265	0.51	5.5	1.4	0.18	0.54
**8**						
MeOH	282	0.61	3.4	0.02	0.29	45.0
CHCl_3_	284	0.36	5.9	0.06	0.17	15.0
PMMA	285	0.25	8.5	2.51	0.12	0.3
PVC	283	0.47	4.6	1.32	0.22	0.5
PS	285	0.29	7.3	0.43	0.14	2.2

*E*_S1_: energy of the singlet state S_1 _ , as 11.96°10^4^ / [( *λ*_A_ + *λ*_F_ )/2], *i.e.*, from the intersection of the normalized fluorescence and absorption spectra, *f*: oscillator strength given as 4.32 ° 10^−9^ ∆*ν*_1/2_*ε*_max_, *τ*_0_: natural radiative lifetime of the excited singlet state, *τ*_F-calc_: calculated fluorescence lifetime as *τ*_F-calc_*=* *τ*_0_Φ_F_, *k*_F-calc_: calculated natural radiative decay rate constant for the excited singlet state as Φ_F_/τ_F-calc_, *k*_nr-cal_: calculated nonradiative decay rate constant for the excited singlet state as (1 − Φ_F_)/τ_F-calc_.

The absorption spectrum of non-substituted coumarin aldehyde **1**, in both the solution and polymer matrices, exhibits two bands without vibrational structure in the UV region at approximately 305 and 350 nm. The absorption maxima (*λ*_A_) of both bands are shifted to longer wavelengths with increasing solvent polarity. The bathochromic shift of absorption maxima implies that the electronic transitions corresponding to these bands are π-π* transitions. Both π-π* transitions are typical for basic coumarin skeletons and are related to the charge transfer from the benzenic cycle to the pyranone moiety [63]. These transitions are associated to an excitation from the HOMO (the highest occupied molecular orbital) and the HOMO-1 orbital, respectively, to the LUMO (the lowest unoccupied molecular orbital). Due to the presence of a relatively strong electron-withdrawing formyl group (CHO) at position 3, the absorption maxima of **1** are shifted more batochromically (~30–40 nm) in comparison with unsubstituted coumarin. Introduction of the methyl substituent to position 7 in the coumarin ring of **1** leads to bathochromic shift of both bands, decreasing the distance between the two absorption bands and changing the ratio of their intensities (Figure 2, compound **5**). The methoxy substitution of hydrogen in position 7 of the coumarin aldehyde **1** promotes an overlapping of these absorption bands to one intense bathochromically shifted absorption band with small long-wavelength shoulder (Figure 2, compound **7**), while the introduction of a dimethylamino group into position 7 of **1** leads to a most intense, bathochromically shifted, single broad structure-less absorption band (Figure 2, compound **3**). This behaviour is explained by the increasing intramolecular charge transfer (ICT) character of molecules **5**,**7** and **3** (in this order, as depicted in Scheme 1), in comparison with the parent carbaldehyde **1**. Because the HOMO-1 to LUMO electronic transition of **1** is (similarly as in the case of unsubstituted coumarin) related to a larger redistribution of charges, the increasing ICT character of compound **5**, **7** and **3** is associated with the decreasing distance and consecutive overlapping of the two absorption bands, increasing intensity (transition dipole moments) and decreasing energy in the corresponding electronic transitions.

The exceptions from the uniform behavior are absorption spectra of aldehydes **1** and **5** in methanol (Supporting Figure 1; Table 1). As shown in Figure S1, these spectra are very different in comparison to spectra of these molecules in chloroform and in all studied polymer matrices (the new absorption band with the highest intensity appears at approximately 280 nm and the ratio of the intensities of two absorption bands above 300 nm alters). They look like the absorption spectra of unsubstituted or 7-methyl-substituted coumarin (with shoulders at approximately 350 nm). This indicates decreased conjugation between the formyl group in position 3 of the coumarin ring and the rest of the molecule. ^1^H-NMR spectra revealed the presence of signal from methoxy protons due to formation of a dimethyl acetal, explaining the changes in the shape of the absorption spectra of **1** and **5**.

The main absorption bands in UV-VIS spectra of phenylsemicarbazides **2**,**4**,**6**,**8** lay also in the near UV region. In comparison to aldehydes **1**,**5**,**7**, the shape of the absorption spectra of phenylsemicarbazides **2**,**6**,**8** changes to one band which is slightly red-shifted to 360–380 nm. We assume that this behaviour is associated with increasing conjugation length of the chromophoric system due to overlapping of π-orbitals of phenylsemicarbazide group with π-orbitals of coumarin moiety. The increasing conjugation length is apparent from the downfield shift of the signal for urea moiety –NH– protons in ^1^H-NMR spectra of semicarbazides **4**,**6** and **8** in comparison to semicarbazide **2** (Supporting Figure 2). As shown in Supporting Figure 2, the introduction of methyl group to position position 7 of the coumarin skeleton leads to remarkable shift of the ^1^H-NMR signal for both –NH– protons of phenylsemicarbazide moiety (the cross-conjugation effect). Condensation of the parent carbaldehydes **1** and **5** with 4-phenylsemicarbazide leads to an increase in the extinction coefficient of semicarbazides **2** and **6**, whereas it only has a weak effect on the extinction of compounds **4** and **8**.

As shown in Figure 2, the substitution with dimethylamino group in position 7 of the coumarin aldehyde **1**, and/or non-substituted semicarbazide **2**, shifts the absorption approximately 80 to 100 nm in both the solution and the polymer matrix, and it also exhibits a higher extinction coefficient (Table 2, compounds **3** and **4**). The methyl and methoxy substitution in position 7 effects a smaller red shift in the longest wavelength band of approximately 10–20 nm. As stated above, this effect is assigned to the increased extent of ICT character in coumarins **3** and **4**, involving coumarins with dimethylamino group in position 7 of the coumarin ring. This is due to the stabilization of resonance structures IV and V depicted in Scheme 1. The hypsochromic shift of *λ*_A_ of **4**, compared to that in the parent aldehyde **3**, in both solution and polymer matrices could thus be a consequence of the weaker electron-withdrawing character of the –C=N-R substituent, compared to the carbonyl group in parent aldehyde **3**.

### 2.2. Fluorescence Spectra

The spectral data based on absorption for parent derivatives **1** and **2**, methyl substituted derivates **5** and **6** and the methoxy substituted **7** are summarized in Table 1. These compounds exhibit zero or weak fluorescence with a quantum yield below 0.001. Substitution in position 7, however, results in a slight increase in fluorescence intensity in both the solution and polymer matrices for these derivatives. The quantum yield value of fluorescence for **5**, **6**, **7** is approximately 0.001. The florescence spectral data for **1**, **2**, **5**, **6**, and **7** exhibits large error, especially for those in the polymer matrices and therefore these are not included in Table 1.

**Figure 3:**
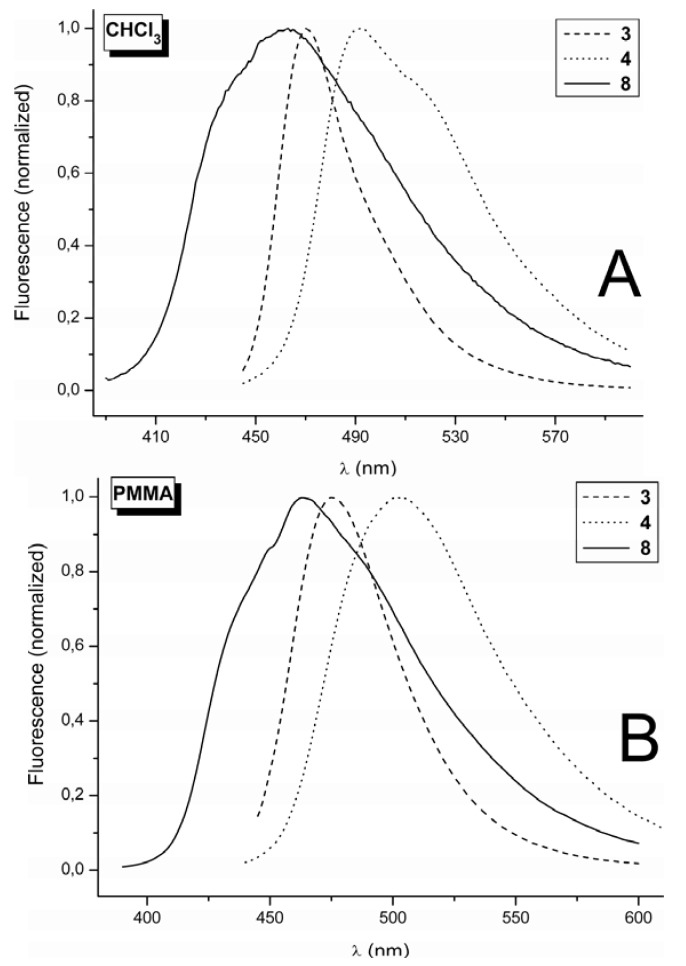
(**A**) Fluorescence spectra of **3**, **4** and **8** in CHCl_3_ at 10^−5^ mol·dm^−3^. (**B**) Fluorescence spectra of **3**, **4** and **8** in PMMA at 0.002 mol kg^−1^.

Introduction of the electron donating group with a +M effect to position 7, as witnessed in the dimethylamino and/or methoxy groups, caused a dramatic increase in the fluorescence intensity, demonstrated in the spectral data for **3** and **4**, and also partly for **8** (Table 2). This effect is generally well documented in the literature [1,5], together with an additional increase in fluorescence intensity after introduction of electron withdrawing group to position 3 of such 7-substituted coumarins [62]. The fluorescence illustrated in Figure 3 consists of a single broad band without vibrational structure. However, for **8** alone, a shoulder is witnessed at the short wavelength edge of the fluorescence band in both the solution and the polymer matrices. A shoulder is also observed for **4** in CHCl_3_, at the long wavelength edge of the fluorescence band (Figure 3A).

The Stoke’s shift, indicating the extent of the red shift of the fluorescence maximum (*λ*_F_) compared to the absorption maxima (*λ*_A_), is the lowest for **3**, falling within the range of 1,500 to 2,500 cm^−1^. Meanwhile, the Stoke’s shift is larger for **4**, at approximately 3,000 cm^−1^, and the largest shift is for **8** at approximately 5,000 cm^−1^. This indicates more significant structural changes between the ground and excited states of **8** compared with those for **4** and **3**. The comparison of *λ*_A_ and *λ*_F_ values for phenylsemicarbazides **4** and **8** (differ only in the character of the substituent in position 7 in the coumarin ring) indicates that the difference in the Stoke’s shift for molecules **4** and **8** results more from the difference in *λ*_A_ than from the difference in *λ*_F_. We assume that the reason for the larger Stoke’s shift of **8** in comparison with **4** is therefore connected with the difference in the ICT character of the ground state of these molecules. The charge redistribution in the ground state of molecule **8** exists somewhere between resonance structures III and IV (Scheme 1), and it is most likely closer to resonance structure IV when this is compared with the charge redistribution in the ground state of **4**.

The intense fluorescence of **3** and **4** exhibits maxima in the 470–505 nm range depending on the medium. Although the determination of quantum yields of fluorescence (Φ) is charged with a large error (of approximately ± 10 up to 20%), the value of Φ is high, reaching 1 for **3** in PVC and for **4** in PMMA and PVC. Previously, high fluorescence was observed for push pull type of di-substituted coumarin with thiosemicarbazide in polar dimethylsulfoxide (DMSO), with the *k*_F_ higher than *k*_nr_, classifying this dye as laser one in the given medium [62]. We observed similar effect for dyes **3** and **4** (Table 2) doped in the polar polymer matrices, where besides decreased rotation of substituent at position 3 some stiffening of the structure of disubstituted coumarin due to dipolar interaction between dopant and matrix may occur. The dyes **3** and **4** might have some potential as laser dyes. The methoxy derivative **8** exhibits lower intensity fluorescence than the **3** and **4** derivatives with 7-dimethylamino substitution.

Comparison of the quantum yields of fluorescence (Φ) of derivatives **3**, **4** and **8** (Table 2) in solution and in polymer matrices reveals that quantum yield values are higher in the polymer matrices than in solution. This effect has already been observed for 3-phenylcoumarin [39]. The decrease in fluorescence is due to the increased rotation of the group in position 3 in the medium of lower micro-viscosity that increases the rate of non-radiative deactivation of the S_1_ state. Comparing Φ within polymer matrices, approximately equal fluorescence quantum-yield values were observed for PMMA and PVC, while PS experienced the lowest values.

The experimentally determined fluorescence lifetimes (τ) are in the range of 0.7–4 ns. Combination of quantum yield Φ and experimentally determined lifetime τ yields the radiation rate constant *k*_F_. This data can be compared with radiation rate constants (*k*_F-calc_), which are calculated using absorption data. This radiation rate constant is expressed as 1/τ_0_, where τ_0_ is the natural radiation lifetime. Comparison of *k*_F_ (Table 2, column 9) and *k*_F-cal_ (Table 3, column 6) shows that *k*_F_ are slightly higher than *k*_F-cal_ for **3**, **4** and **8**. Both *k*_F_ and *k*_F-cal_ are in the range of 1–10 × 10^8^ s^−1^. Some discrepancies are noted, however, for fluorophor **3** in the PS matrix. The quantum yields of all three chromophores **3**, **4** and **8** in the PS polymer matrix are lower compared to those in PMMA and PVC. At the same time, *k*_F_ is one order lower than the *k*_F-calc_ for **3** in PS. This data clearly indicates that the PS matrix is less inert than PMMA and PVC, and that it exerts a quenching effect on the studied push-pull coumarins. The PMMA and PVC matrices appear to have the required polarity and micro-viscosity to suppress non-radiative processes, so that the quantum yield of fluorescence approaches unity in these matrices. We assume that specific π-π interactions between coumarin moiety of the studied compounds and benzene ring of the PS matrix contribute to non-radiative decay of the excited states of the studied coumarins. Futher studies should determine whether any additional factors play a role in these mechanisms.

## 3. Experimental

### 3.1. General

Melting points (uncorrected) were measured on a Kofler hot stage. ^1^H- and ^13^C-NMR spectra were recorded on Varian Mercury Plus 300 spectrometer (operating at 300 MHz for ^1^H and 75 MHz for ^13^C) in DMSO-d_6_ or CDCl_3_ with TMS as internal standard. Chemical shifts (*δ*) are reported in ppm downfield of TMS and coupling constants (*J*) are expressed in Hertz (Hz). All the chemicals and solvents were purchased from major chemical suppliers (Merck, Darmstadt, Germany; Acros Organics, Geel, Belgium; Sigma-Aldrich, St. Louis, MO, USA) in highest grade purity, and all solvents were dried by standard methods and distilled prior to use. Elemental analyses were performed on a Carlo Erba Strumentacione 1106 apparatus. 7-(Dimethylamino)-2-oxo-2*H*-chromene-3-carbaldehyde (Mp 204–205 °C, lit. [60] 205 °C) was prepared by 3-step reaction, according to literature [60]. 7-H (Mp 133–134 °C, lit. [64] 134 °C), 7-methyl (Mp 174–176 °C, lit. [64] 176 °C) and 7-methoxy (Mp 235–237 °C, lit. [64] 238 °C) carbaldehydes were prepared by a modified procedure [64,65]. The structure of prepared aldehydes was proved from their ^1^H-NMR spectra.

### 3.2. Synthesis of Coumarin Derivatives

**Scheme 2:**
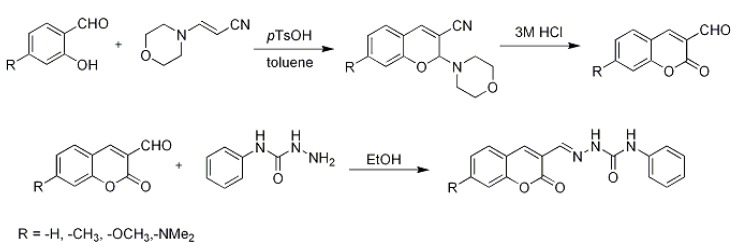
Synthesis of substituted coumarins.

The general synthetic route is shown in Scheme 2.

### 3.3. Reaction of Aldehydes with Phenylsemicarbazide

General Procedure

A solution of phenylsemicarbazide (0.7 mmol; 105 mg) in hot absolute ethanol (3 mL) was added to 2-oxo-2*H*-chromene-3-carbaldehyde (0.7 mmol) in hot absolute EtOH (5 mL). The reaction mixture was refluxed for 15 min, and the desired products was precipitated, filtered and washed with cold ethanol, and then dried and re-crystallized from ethanol. The crude products were obtained in 98–99% yields.

*(*E*)-1-[(2-Oxo-2*H*-chromen-3-yl)methylidene]-4-phenylsemicarbazide* (**2**): Obtained from **1 **(122 mg) in 97% yield (208 mg), Mp 216–219 °C, ^1^H-NMR (CDCl_3_): δ 7.03–7.07 (1H, *m*, Ar-H), 7.30–7.35 (2H, *m*, Ar-H), 7.39–7.47 (2H, *m*, Ar-H), 7.62–7.66 (3H, *m*, Ar-H), 7.77 (1H, *dd*, *J* = 7.8 Hz, 1.5 Hz, H-8), 8.05 (1H, *s*, H-4), 8.86 (1H, *s*, NH), 9.01 (1H, *s*, NH), 11.10 (1H, *s*, HC=N); ^13^C-NMR (CDCl_3_): δ 116.29, 119.19, 120.13, 121.299, 122.78, 125.04, 128.51, 128.92, 132.18, 133.69, 137.24, 138.82, 152.75, 153.09, 159.88; Anal. Calcd for C_17_H_13_N_3_O_3_ (307.3): C, 66.44; H, 4.26; N, 13.68. Found: C, 66.42; H, 4.33; N 13.65. 

*(*E*)-1-{[(7-Dimethylamino)2-oxo-2*H*-chromen-3-yl]methylidene}-4-phenylsemicarbazide* (**4**): Obtained from **3** (152 mg) in 98% yield (240 mg), ^1^H-NMR (DMSO): δ 3.07 (6H, *s*, -N(CH_3_)_2_), 6.61 (1H, *d*, *J* = 2.1 Hz, Ar-H), 6.81 (1H, *dd*, *J* = 9 Hz, 2.1 Hz, Ar-H), 7.00–7.05 (1H, *m*, Ar-H), 7.28–7.33 (2H, *m*, Ar-H), 7.53 (1H, *d*, *J* = 9 Hz, Ar-H), 7.66 (2H, *dd*, *J* = 8.7 Hz, 1.2 Hz, Ar-H), 7.99 (1H, *s*, H-4), 8.68 (1H, *s*, NH), 8.89 (1H, *s*, NH), 10.85 (1H, *s*, HC=N); ^13^C-NMR (DMSO): δ 40.35, 97.17, 108.49, 110.06, 113.45, 119.89, 122.54, 128.47, 129.85, 135.01, 138.47, 129.00, 152.85, 153.24, 155.81, 160.74; Anal. Calcd for C_19_H_18_N_4_O_3_ (350.4): C, 65.13; H, 5.18; N, 15.99. Found: C, 65.12; H, 5.14; N 15.82.

*(*E*)-1-[(7-Methyl-2-oxo-2*H*-chromen-3-yl)methylidene]-4-phenylsemicarbazide* (**6**): Obtained from **5 **(132 mg) in 98% yield (220 mg), Mp 217–218 °C ^1^H-NMR (CDCl_3_): δ 2.49 (3H, s, CH_3_), 7.09–7.19 (3H, *m*, Ar-H), 7.33–7.39 (2H, *m*, Ar-H), 7.50–7.58 (3H, *m*, Ar-H), 7.95 (1H, *s*, H-4), 8.04 (1H, *s*, NH), 8.23(1H, *s*, HC=N), 8.29 (1H, *s*, NH); ^13^C-NMR (CDCl_3_) δ 22.01, 116.56, 117.04, 119.76, 119.82, 123.83, 126.33, 128.40, 129.08, 135.07, 137.53, 137.78, 144.33, 152.20, 153.93, 160.35; Anal. Calcd for C_18_H_15_N_3_O_3_ (321.3): C, 67.28; H, 4.71; N, 13.08. Found: C, 67.43; H, 4.72; N 13.07.

*(*E*)-1-[(7-Methoxy-2-oxo-2*H*-chromen-3-yl)methylidene]-4-phenylsemicarbazide* (**8**): Obtained from **7** (143 mg) in 97% yield (229 mg), Mp 218–220 °C ^1^H-NMR (CDCl_3_): δ 3.91 (3H, s, -OCH_3_), 6.86 (1H, *d*, *J* = 2.4 Hz, Ar-H), 6.92 (1H, *dd*, *J* = 8.7 Hz, 2.4 Hz, Ar-H), 7.08–7.14 (1H, *m*, Ar-H), 7.33–7.39 (2H, *m*, Ar-H), 7.51–7.60 (3H, *m*, Ar-H), 7.92 (1H, *s*, H-4), 8.03 (1H, *s*, NH), 8.09 (1H, *s*, NH), 8.22 (1H, *s*, HC=N); ^13^C-NMR (CDCl_3_): δ 55.96, 100.79, 103.98, 112.61, 113.60, 117.37, 119.75, 123.79, 129.07, 129.79, 135.23, 137.57, 137.99, 152.13, 155.86, 163.71; Anal. Calcd for C_18_H_15_N_3_O_4_ (337.3): C, 64.09; H, 4.48; N, 12.46. Found: C, 64.08; H, 4.51; N 12.29.

### 3.4. Spectral Measurements

Anthracene was zonally refined (Lachema n.e., Brno, CR, USA). The methanol and chloroform were UV spectroscopy grade (Merck, Darmstadt, Germany). Polymer films doped with coumarins were prepared by casting from solution. Films of polystyrene (PS; Kratsen, Kaucuk s.e., Kralupy, ČR, M_w_ = 1.1 × 10^5^ D) and poly(methylmethacrylate) (PMMA; Diacon, ICI, England, M_v_ = 1.01 × 10^5^ D) were prepared by casting 1 mL chloroform solution of polymer (5 g/100 mL) containing the appropriate amount of probe onto a 28 × 35 mm glass plate. The solvent was evaporated slowly. Films of poly(vinylchloride) (PVC) (Neralite 628, Spolana Neratovice, s.e., ČR, M_w_ = 1.11 × 10^5^ D) were prepared similarly by casting from tetrahydrofuran solution. All three polymers were additive free. The rest of solvents were additionally not removed from the polymer.

UV-VIS absorption spectra were recorded with an UV 1650PC spectrometer (Shimadzu, Japan), and fluorescence spectra with either a RF-5301 (Shimadzu, Japan) or a FSP 920 (Edinburgh Instruments, UK) spectrofluorimeters. The Origin 6.1 (Microcal) was used for data plotting. Fluorescence of solution was measured in a 1 cm cuvette in the right-angle arrangement, and the quantum yields were determined relative to anthracene in chloroform and methanol. Polymer film fluorescence was taken in front face arrangement on the solid sample holder.

The fluorescent quantum yield of disubstituted coumarins was determined in solution and in polymer films using anthracene as the standard in the given medium, taking the quantum yield of anthracene in cyclohexane equal to 0.25 [66]. The anthracene fluorescence quantum yields in the different media were determined by the comparison with the anthracene fluorescence in cyclohexane. These values are 0.22 in acetonitrile, 0.20 in methanol, and 0.11 in chloroform. In polymer matrices, the quantum yields were assumed to be 0.20 in PMMA, 0.16 in PS and 0.11 in PVC. The quantum yields in the solutions and films were corrected to different absorptions at the excitation wavelength [67], and fluorescence spectra were taken using excitation at the longest wavelength absorption-band maxima.

The fluorescence lifetime measurements were performed on a LIF 200 (Lasertechnik Ltd., Berlin, Germany), which operates as a stroboscope. The excitation source was a nitrogen laser emitting at 337 nm and the emission was selected by cut-off filter. The output signal of Box Car Integrator was digitized and transferred to the PC using a home-made programme. The fluorescence decay curves were evaluated by the simple phase plane method [68] using J. Snyder’s programme [69]. The standard deviation G^1/2^ = Σ((I_exp_ − I_calc_)^2^/n)^1/2^, where I_exp_ and I_calc_ define the intensities of experimental and calculated emission, respectively, was employed to establish if the decay was mono-exponential. It was assumed that the decay curve satisfies the mono-exponential when G^1/2^ was below 5%. The fluorescence decay of the studied compounds was reasonably fitted with monoexponential function. The error was less than 5%. The steady state and time-resolved fluorescence measurements were performed in aerated solutions. All measurements on polymer films were performed in the air.

### 3.5. Relationships in the Calculation of Spectral Properties


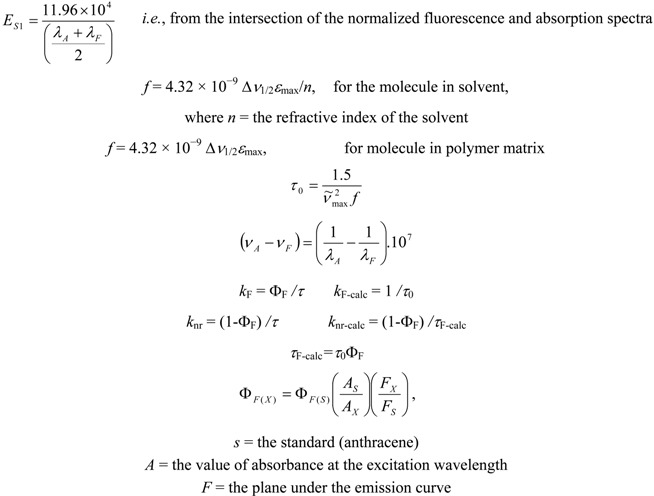


## 4. Conclusions

Substituted coumarins (2-oxo-2*H*-chromenes) with various electron donating ability in position 7, such as H, CH_3_, OCH_3_, N(CH_3_)_2_ and substituted in position 3 by CHO or –CH=NNHCONHPh, were investigated. Introduction of the electron-donating group to position 7 of the coumarin ring in the non-substituted coumarin aldehyde leads to a noticeable change in absorption spectrum shape and a substantial red-shift of the absorption maximum. This behaviour is explained by the increasing intramolecular charge transfer (ICT) character of substituted aldehydes. Replacement of the formyl group in position 3 of the coumarin ring by a –CH=NNHCONHPh group also markedly influences the shape of the absorption spectrum, and this change is substantial in non-substituted and methyl-substituted coumarins. We assume that increasing conjugation length of the chromophoric system (due to overlapping of π-orbitals of phenylsemicarbazide group with π-orbitals of coumarin moiety) is responsible for this effect. However, only a small change was recorded in the 7-dimethylamino substituted coumarin. 

The fluorescence is almost negligible for derivatives not substituted in position 7 (compounds **1** and **2**), and also for derivatives with weak or medium electro-donating character of substituent in position 7 (compounds **5**, **6** and **7**). Introduction of a strong electron donating group with +M effect, such as the dimethylamino group, to position 7 of the coumarin ring provides a significant increase in fluorescence intensity. A noticeable fluorescence was observed for the methoxy derivative **8**, and it was very intense in the 7-dimethylamino coumarins **3** and **4**. Surprisingly, the different charge redistribution in the ground state of molecules **3**,**4** and **8** is mainly responsible for the different values of the Stoke’s shifts of these molecules. The quantum yield of both 7-dimethylamino derivatives approaches 1 in PMMA and PVC polymer matrices. The finding that chromophores **3** and **4** exhibit quantum yields approaching 1 in the polymer matrices is explained by the medium-suppressed rotation of the side substituent at position 3 in the given media. We assume that stiffening of the dopant due to higher micro-viscosity and dipole-dipole interaction between dopant and matrix occurs in the polar matrices of PMMA and PVC. The dyes 3 and 4 might be classified as laser ones in the given medium. The fluorescence lifetime is in the range 0.5–4 ns. Based on experimental results, the fluorescence radiation rate constant is approximately 5 × 10^8^ s^−1^, and this value is in accordance with those determined from the absorption spectra.

## Supplementary Materials

Supplementary materials can be accessed at: http://www.mdpi.com/1420-3049/17/3/3259/s1.
