# Supplementary material for: Spectral Properties of Substituted Coumarins in Solution and Polymer Matrices
Source: Molecules. 2012 Mar 14;17(3):3259–76. doi: 10.3390/molecules17033259 (PMC6268095; doi:10.3390/molecules17033259)

# Supporting Information

# Spectral Properties of Substituted Coumarins in Solution and Polymer Matrices

**Jana Donovalová [1,*], Marek Cigáň [1], Henrieta Stankovičová [1], Jan Gašpar [1], Martin Danko [2], Anton Gáplovský [1] and Pavol Hrdlovič [1,2]**

[1] Faculty of Natural Sciences, Institute of Chemistry, Comenius University, Mlynská dolina CH-2, SK-842 15 Bratislava, Slovak; E-Mails: cigan@fns.uniba.sk (M.C.); stankovh@fns.uniba.sk (H.S.); gaspar@fns.uniba.sk (J.G.); gaplovsky@fns.uniba.sk (A.G.)

[2] Polymer Institute, Slovak Academy of Sciences, 842 36 Bratislava, Dúbravská cesta 9, Slovak; E-Mails: upoldan@savba.sk (M.D.); upolhrdl@savba.sk (P.H.)

* Author to whom correspondence should be addressed; E-Mail: donovalova@fns.uniba.sk; Tel.: +421-2-6029-6306; Fax: +421-2-6029-6337.

**Supporting Figure 1.** Absorption spectra of carbaldehydes **1** and **5** in chloroform and methanol at ~$10^{-4}$ mol dm$^{-3}$.

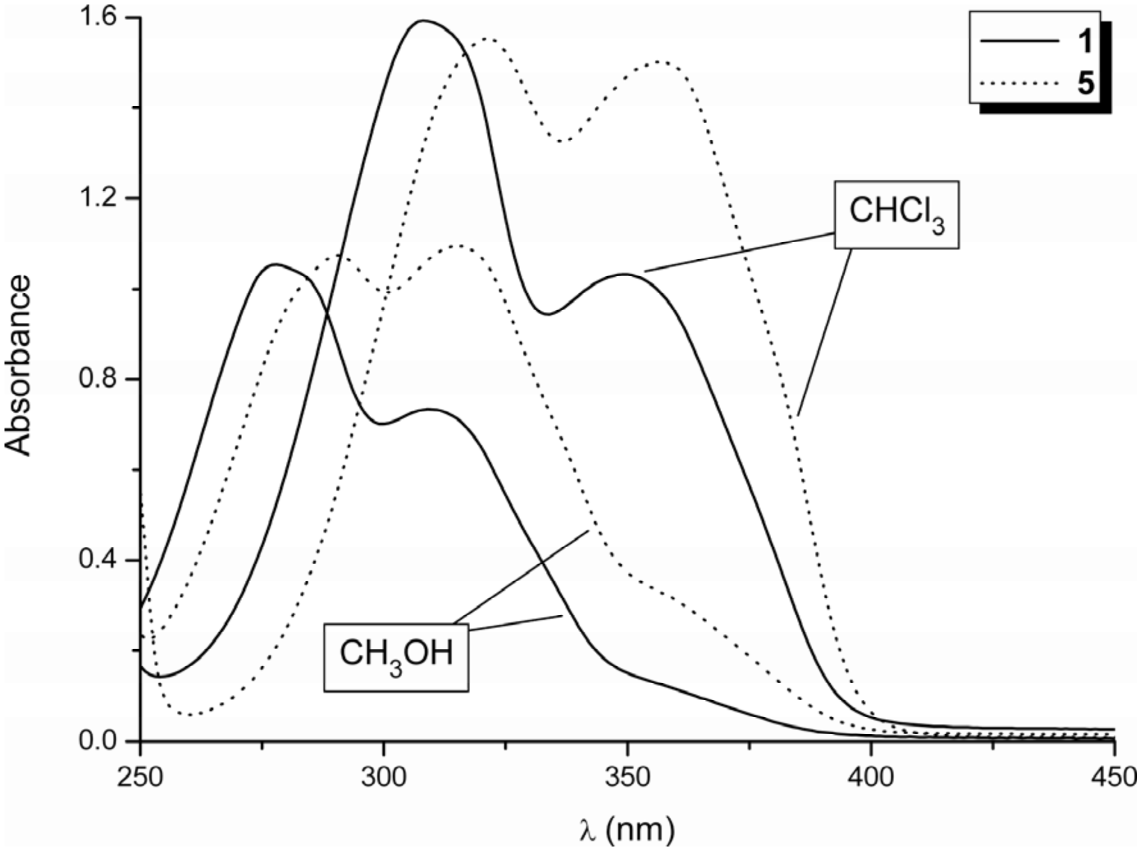

**Supporting Figure 2.** The change in chemical shift of signals for various protons of phenylsemicarbazide group for investigated 7-substituted phenylsemicarbazides **4**,**6**,**8** and the basic 7-unsubstituted phenylsemicarbazide **2**.

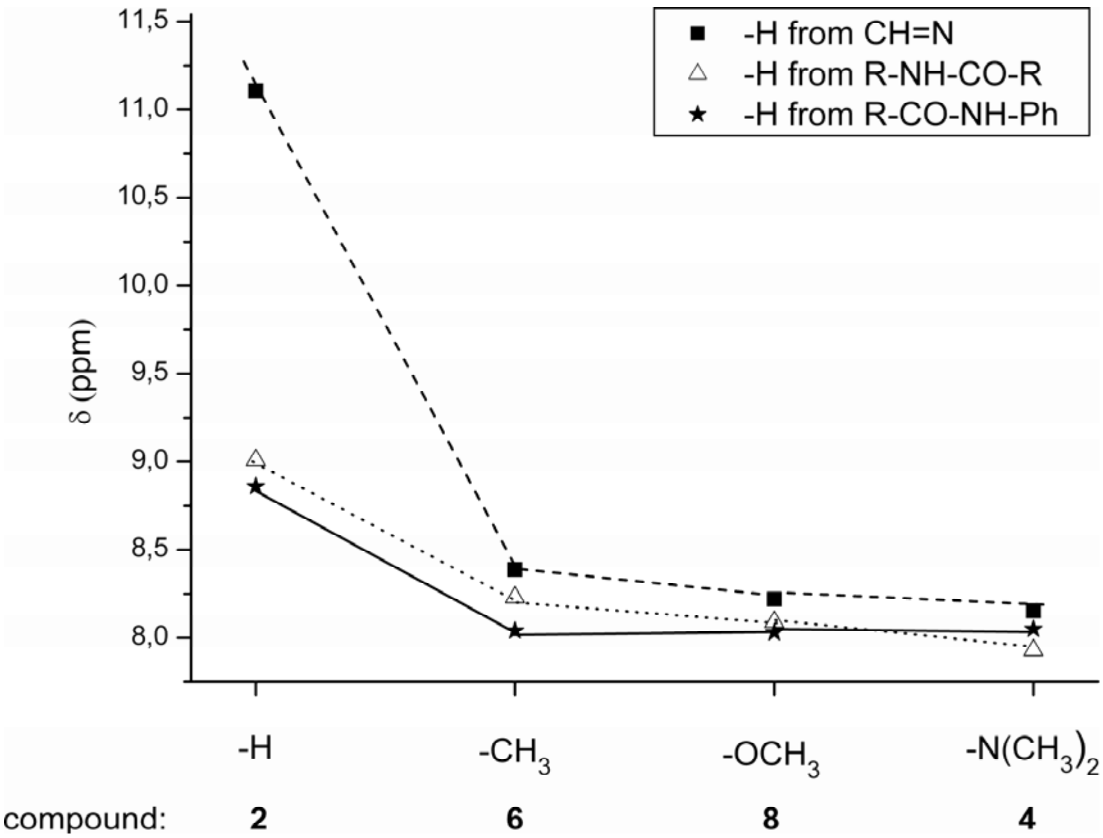

Supplement: Supplementary file 1 [file molecules-17-03259-s001.pdf]
